# Determinants of maternal near-miss among women admitted to public hospitals in North Shewa Zone, Ethiopia: A case-control study

**DOI:** 10.3389/fpubh.2022.996885

**Published:** 2022-08-25

**Authors:** Hana Nigussie Teshome, Esubalew Tesfahun Ayele, Solomon Hailemeskel, Osman Yimer, Getaneh Baye Mulu, Mesfin Tadese

**Affiliations:** ^1^Department of Nursing, College of Health Sciences, Debre Berhan University, Debre Berhan, Ethiopia; ^2^Department of Public Health, College of Health Sciences, Debre Berhan University, Debre Berhan, Ethiopia; ^3^Department of Midwifery, College of Health Sciences, Debre Berhan University, Debre Berhan, Ethiopia

**Keywords:** maternal near-miss, determinants, North Shewa, Ethiopia, case-control study

## Abstract

**Background:**

A maternal near-miss (MNM) refers to a woman who presents with life-threatening complications during pregnancy, childbirth, or within 42 days of termination of pregnancy but survived by chance or due to the standard care she received. It is recognized as a valuable indicator to examine the quality of obstetrics care as it follows similar predictors with maternal death. Ethiopia is one of the sub-Saharan African countries with the highest rate of maternal mortality and morbidity. Thus, studying the cause and predictors of maternal near-miss is vital to improving the quality of obstetric care, particularly in low-income countries.

**Objective:**

To identify determinants of maternal near-miss among women admitted to public hospitals in North Shewa Zone, Ethiopia, 2020.

**Methods:**

A facility-based unmatched case-control study was conducted on 264 women (88 cases and 176 controls) from February to April 2020. Data were collected using pretested interviewer-administered questionnaires and a review of medical records. Data were entered into Epi-data version 4.2.2 and exported to SPSS version 25 for analysis. Variables with a *p*-value <0.25 in the bivariable analysis were further analyzed using multivariable logistic regression analysis. Finally, variables with a *p*-value <0.05 were considered statistically significant.

**Result:**

Severe pre-eclampsia (49.5%) and postpartum hemorrhage (28.3%) were the main reasons for admission of cases. Educational level of women (AOR = 4.80, 95% CI: 1.78–12.90), education level of husbands (AOR = 5.26; 95% CI: 1.46–18.90), being referred from other health facilities (AOR = 4.73, 95% CI: 1.78–12.55), antenatal care visit (AOR = 2.75, 95% CI: 1.13–6.72), cesarean section (AOR = 3.70, 95% CI: 1.42-9.60), and medical disorder during pregnancy (AOR = 12.06, 95% CI: 2.82–51.55) were found to significantly increase the risk of maternal near-miss. Whereas, the younger age of women significantly decreased the risk of maternal near miss (AOR = 0.26, 95% CI: 0.09–0.75).

**Conclusion:**

Age, educational level, antenatal care follow-ups, medical disorder during pregnancy, mode of admission, and mode of delivery were significant predictors of maternal near-miss. Socio-demographic development, use of ANC services, early detection and management of medical diseases, reducing cesarean section, and improving the referral systems are crucial to minimizing the maternal near-miss.

## Introduction

According to World Health Organization (WHO), maternal near-miss occurs when women present with life-threatening complications during pregnancy, childbirth, or within 42 days of termination of pregnancy but survived by chance or because they received care in health facilities (1). In Sustainable Development Goals (SDG), improving maternal health remains an important issue, which was planned to reduce the global Maternal Mortality Ratio (MMR) to less than 70 per 100,000 live births by the year 2030 (2). However, maternal mortality remains unacceptably high, particularly in low-income countries, in which 99% of maternal death occurs. The risk of maternal death is one in 41 and one in 3300 live births in developing and developed countries, respectively (3). Sub-Saharan Africa alone contributes approximately 66%, followed by Southern Asia, 22% of maternal death (4). In explaining this disparity, access to quality care during pregnancy, childbirth, and the postnatal period was critical (3, 5).

Ethiopia is one of the Sub-Saharan African countries with the highest rate of maternal mortality. Based on the Ethiopian Demographic and Health Survey (EDHS) report, maternal mortality declined from 1400 in 1990 to 676 per 100,000 live births in 2011, and the 2016 EDHS documented 412 deaths per 100,000 live births (6, 7). According to the National Maternal and Perinatal Death Surveillance and Response (MPDSR) System Annual Report of 2012 E.C, the leading causes of maternal morbidity and mortality were hemorrhage (37%), hypertensive disorders of pregnancy (HDP) (11%), anemia (16%), and sepsis (6%) (8).

To reduce maternal morbidity and mortality, the country has taken different actions to improve basic emergency care for obstetric cases. These include improving the quality of antenatal care (ANC) in a health center, improving access to blood transfusion at a primary hospital, expansion of comprehensive emergency obstetric and newborn care (CEmONC) health facilities, strengthening catchment-based mentorship program, strengthening the referral system, availing anticonvulsant treatment and uterotonic drugs and commodities including non-pneumatic anti-shock garment (NASG) and monitoring of severe hypertension disorder of pregnancy (HDP) at a referral hospital and improve access for ambulance service (8). Despite all these measures, there is still a significant obstacle in terms of the provision of quality obstetrics care. According to EDHS 2019, only 50% of women were delivered by a skilled provider, and only 48% were delivered in a health facility (9).

Maternal near-miss surveillance is helpful to identify subgroups of risk that require special health care and resource allocation. Women who survived life-threatening complications related to pregnancy and childbirth have many common aspects with those who die of such complications. Exploring women who survived life-threatening conditions provides a complete assessment of the quality of maternal health care (10).

Maternal near-miss remains a significant public health problem in both developed and undeveloped countries. According to a recent systematic review and meta-analysis study, the global prevalence of maternal near miss was 1.4% (11). Studies in Nigeria (12), Tanzania (13), and Ethiopia (14) reported 198 per 1000, 87.4 per 1000 live births, and 12.57% prevalence of MNM, respectively. Rural residents, low level of education, being unmarried, lack of antenatal care, history of stillbirth, referred cases, and far distance of health institutions had a statistically significant association with MNM (14, 15).

Maternal death accounts small percentage of maternal morbidity, only the tip of the iceberg. Despite a decrease in maternal mortality rate, the morbidity rate remains elevated. To further reduce maternal death at the grass-root level, life-threatening severe maternal factors contributing to MNM/ severe maternal morbidities need to be addressed. It is, therefore, necessary to investigate the extent of all this morbidity and predict factors that expedite turning it into mortality. In addition, assessing and studying those mothers, who escaped from being treated as dead, is essential to reduce the future risk of death and improve maternal health. However, limited studies have been done to explore those at-risk mothers in Northern Ethiopia. Therefore, the study aimed to investigate the determinants of maternal near-miss among women admitted to the selected public hospitals of the North Shewa Zone, Ethiopia.

## Methods

### Study area and period

A hospital-based unmatched case-control study was conducted in North Shewa Zone public hospitals from February 1 to April 30, 2020. North Shewa is one of the 13 zones found in Amhara regional state, with the administrative center of Debre Berhan town located 130 km from Addis Ababa (the capital city of Ethiopia). The zone has 24 woredas and four city administrations. North Shewa Zone has an estimated total population of 2.3 million. Women aged 15–49 were estimated to be 20.23%, and the estimated number of pregnant women is around 77,483. The zone has one referral hospital, nine primary hospitals, and 196 health centers.

### Operational definition

**Maternal near-miss:** When a woman faced either of the following WHO criteria but survived. (1) Severe maternal complications (Severe postpartum hemorrhage, Severe pre-eclampsia, Eclampsia, Sepsis/severe systemic infection, or ruptured uterus). (2) Provision of critical intervention (Admission to intensive care unit, Blood transfusion, Interventional radiology, and Laparotomy including hysterectomy and other emergency surgical interventions in the abdominal cavity, but excluding cesarean section. (3) Organ dysfunction, i.e., Cardiovascular, Respiratory, Renal, Coagulation/hematological, Hepatic, Neurological, and Uterine dysfunction (1).

### Population and eligibility criteria

All mothers admitted to the study hospitals during pregnancy, childbirth, or within 42 days after giving birth were the source population. Women who fulfilled the WHO near-miss criteria were selected as cases. Women who were admitted for normal vaginal delivery or who did not have any of the complications mentioned in the WHO near-miss criteria were considered controls.

Mothers who were initially selected as control and discharged but unfortunately returned as a case and those mothers who were unable to communicate or seriously ill during data collection were excluded from the study.

### Sample size determination and sampling procedure

The sample size was calculated using Epi-info version 7.2.0.1 statistical software. Considering the percent of controls exposed (Age at first pregnancy) 18.5%, odds ratio 2.5 (16), 95% confidence interval, power 80%, and the ratio of cases to controls two. Adding a 10% non-response rate, the final sample size was 264 (88 cases and 176 controls).

Three public hospitals (Debre Berhan Referral Hospital, Enat Hospital, and Ataye Hospital) were selected by lottery method from ten public hospitals in North Shewa. The proportional allocation was made for each hospital by fixing a one-month average delivery flow. Then, cases were selected by consecutive sampling. For each case, two controls were selected from the same hospitals from which cases were drawn.

### Data collection tool and measurement

The data were collected through face-to-face interviews and a review of medical records. It was conducted after the mother's condition was improved and assessed to be capable of responding to the interview. Five trained midwives and two supervisors participated in the data collection. The questionnaire was adapted from the WHO and other peer-reviewed articles (1, 16, 17). The questionnaire contains four sections. The first section included the socio-demographic characteristics of the study subjects. The second part addresses current and previous obstetric history. The third section contained medical disorders of pregnancy, and the final part consisted of the three delays for maternal health care services.

### Data quality control

The questionnaire was first prepared in English and translated to the local language, Amharic, and then back to English to check for language consistency. A pre-tested was done on 5% of the sample size before actual data collection. A necessary amendment was made following the result of the pre-test. The investigators trained data collectors and supervisors about the objective, data collection tool, and procedure for one day. The investigators and supervisors have closely supervised, checked, and reviewed the data for clarity, completeness, and consistency. Consistency was assessed through a random selection of questionnaires.

### Data processing and analysis

The data were cleaned, coded, and entered into Epi-Data version 4.2.2 and exported to SPSS version 25 for analysis. Descriptive statistics like frequency, distribution, and summary statistics such as mean and standard deviation were computed for cases and control groups. For smaller expected frequencies, merge of variables was made. An independent sample t-test was used to test the equality of means for selected continuous variables among cases and controls. Bivariable and multivariable logistic regression analysis models were run to identify independent determinants of maternal near-miss. Variables with a *p*-value of ≤0.25 in the bivariable regression models were candidates for the multivariable logistic regression analysis models. The level of multicollinearity was checked and fitted using variance inflation factor and tolerance and found within a tolerable range. The model's goodness of fit, Hosmer Lemeshow, was checked and fitted for the data. A *p*-value of <0.05 was considered statistically significant.

## Result

### Socio-demographic characteristics

A total of 264 participants were interviewed, making a 100% response rate. The mean (±SD) age of cases and controls was 30.4+ 5.6 and 29.6 + 4.6 years. Participants aged 25–34 years accounted for 54(61.3%) of cases and 122(69.4%) of controls. Sixty-two (70.5%) of cases and one hundred two (58%) of controls were rural residents. Thirty-six (40.9%) of cases had no formal education, while one hundred one (57.4%) of controls had attended above secondary school. Besides, 48.9% of cases and 14.2% of controls had less than 2500-birr monthly income (Table 1).

**Table 1:** Socio-demographic characteristics of mothers admitted to public hospitals of North Shewa Zone, Ethiopia, 2020 (N = 264).

**Variable**	**Case *n* (%)**	**Control *n* (%)**	**Total *n* (%)**
**Age**			
15–24	13(14.8)	30(17.0)	43(16.3)
25–34	54(61.3)	122(69.4)	176(66.7)
≥35	21(23.9)	24(13.6)	45(17.0)
**Residence**			
Urban	26(29.5)	74(42)	100(37.9)
Rural	62(70.5)	102(58)	164(62.1)
**Educational status of women**			
No formal education	36(40.9)	29(16.5)	65(24.6)
Primary & secondary	35(39.8)	46(26.1)	81(30.7)
Above secondary	17(19.3)	101(57.4)	118(44.7)
**Mother occupation**			
Housewife	42(47.7)	35(19.9)	77(29.2)
Merchant	9(10.3)	19(10.8)	28(10.6)
Employee	37(42)	122(69.3)	159(60.2)
**Marital status**			
Married	86(97.7)	166(94.3)	252(95.5)
Single	2(2.3)	10(5.7)	12(4.5)
**Educational status of husband**			
No formal education	39(45.3)	23(13.7)	62(24.4)
Primary & secondary	28(32.6)	49(29.2)	77(30.3)
Above secondary	19(22.1)	96(57.1)	115(45.3)
**Occupation of husband**			
Farmer	39(45.3)	20(11.9)	59(23.2)
Merchant	17(19.8)	26(11.5)	43(16.9)
Employee	30(34.9)	122(72.6)	152(59.8)
**Income (in ETB)**			
<2500	43(48.9)	25(14.2)	68(25.8)
2501–3850	27(30.7)	37(21.0)	64(24.2)
3851–6000	16(18.2)	60(34.1)	76(28.8)
>6000	2(2.3)	54(30.7)	56(21.2)

ETB, Ethiopian Birr.

### Reproductive health and obstetrics history

About 8(9.1%) of cases were married before the age of 18 years as compared to 23(13.1%) of controls and 40(45.5%) of cases had their first pregnancy before the age of 20 years when compared to 33(18.8%) of controls. Regarding the number of pregnancies, 80(90.9%) of cases and 152(86.4%) of controls were multigravidas. Five (6.2%) of cases and twenty-one (13.8%) of controls had a history of abortion. Besides, more than half of the cases, 55(62.5%) and controls 148(84.1%), had received antenatal care (ANC) for the current pregnancy (Table 2).

**Table 2:** Obstetrics history among mothers admitted to public hospitals of North Shewa Zone, Ethiopia, 2020 (N = 264).

**Variable**	**Case *n* (%)**	**Control *n* (%)**	**Total *n* (%)**
**Age at marriage**			
< 18 years	8(9.1)	23(13.1)	31(11.7)
>19 years	80(80.9)	153(86.9)	233(88.3)
**Age at first pregnancy**			
<20 years	40(45.5)	33(18.8)	73(27.7)
> 21 years	48(54.5)	143(81.2)	191(72.3)
**Number of pregnancies**			
Primigravida	8(9.1)	24(13.6)	32(12.1)
Multigravida	80(90.9)	152(86.4)	232(87.9)
**Antenatal care visit**			
Yes	55(62.5)	148(84.1)	203(76.9)
No	33(37.5)	28(15.9)	61(23.1)
**History of Abortion**			
Yes	5(6.2)	21(13.8)	26(11.2)
No	75(93.8)	131(86.2)	206(88.8)
**Pregnancy-related complication**			
Yes	19(23.8)	18(11.8)	37(15.9)
No	61(76.2)	134(88.2)	195(84.1)
**Antepartum hemorrhage (APH)**			
Yes	3(15.8)	4(22.2)	7(18.9)
No	16(84.2)	14(77.8)	30(81.1)
**Pregnancy-induced HTN**			
Yes	7(36.8)	7(38.9)	14(37.8)
No	12(63.2)	11(61.1)	23(62.2)
**Pre-eclampsia/ eclampsia**			
Yes	2(10.5)	1(5.6)	3(8.1)
No	17(89.5)	17(94.4)	34(91.9)
**Postpartum hemorrhage (PPH)**			
Yes	4(21.1)	6(33.3)	10(27)
No	15(78.9)	12(66.7)	27(73)
**Obstructed labor**			
Yes	4(21.1)	3(16.7)	7(18.9)
No	15(78.9)	15(83.3)	30(81.1)
**Mode of delivery in last pregnancy**			
Spontaneous vaginal delivery	62(77.5)	138(90.8)	200(86.2)
Cesarean section (C/S)	18(22.5)	14(9.2)	32(13.8)
**Birth interval**			
Short (< 23 months)	44(55.0)	50(32.9)	94(40.5)
Normal (24–60 months)	36(45.0)	102(67.1)	138(595)

### Medical disorder of pregnancy

Fourteen (15.9%) of cases and eight (4.5%) of controls had a history of medical disorder during pregnancy. Of these, anemia was reported in 10(71.4%) of cases and 6(75.0%) of controls (Table 3).

**Table 3:** Medical disorder of pregnancy among mothers admitted to public hospitals of North Shewa Zone, Ethiopia, 2020 (N = 246).

**Variable**	**Case *n* (%)**	**Control *n* (%)**	**Total *N* (%)**
**Medical disorder**			
Yes	14(15.9)	8(4.5)	22(8.3)
No	74(84.1)	168(95.5)	242(91.7)
**Anemia**			
Yes	10(71.4)	6(75.0)	16(72.7)
No	4(28.6)	2(25.0)	6(27.3)
**Gestational diabetes**			
Yes	1(7.1)	1(12.5)	2(9.1)
No	13(92.9)	7(87.5)	20(90.9)
**Renal disease**			
Yes	2(14.3)	1(12.5)	3(13.6)
No	12(85.7)	7(87.5)	19(86.4)
**Pneumonia**			
Yes	1(7.1)	0(0)	1(4.5)
No	13(92.1)	8(100)	21(95.5)

### Clinical characteristics of maternal near-miss

Maternal near-miss cases were admitted with various primary diagnoses. The most common causes of maternal near-miss were severe pre-eclampsia (49.5%) followed by postpartum hemorrhage (28.3%), eclampsia (14.1%), sepsis (6.1%), and uterine rupture (2.0%).

### Maternal health service-related characteristics

Forty-three (48.9%) of cases were referred from other health facilities compared to thirty-two (18.2%) of controls. About thirty-four (38.6%) of cases and thirty-seven (21.0%) of controls had reported delays in decision-making to come to the hospital. Failure to understand the problem (63.6%) was the main reason for the delay among cases while underestimating the severity of the condition (37.8%) was the prominent reason among controls (Table 4).

**Table 4:** Patterns of the three delays for maternal health care among mothers admitted to public hospitals of North Shewa Zone, Ethiopia, 2020 (N = 264).

**Variable**	**Case**	**Control**	**Total**
	***n* (%)**	***n* (%)**	***n* (%)**
**Mode of admission**			
Self	45(51.1)	144(81.8)	189(71.6)
Refer to other health facilities	43(48.9)	32(18.2)	75(28.4)
**Responsible for decision making**			
Self	55(62.5)	97(55.1)	152(57.6)
Husband	27(30.7)	75(42.6)	102(38.6)
Relatives	6(6.8)	4(2.3)	10(3.8)
**Delay to decide to come to the hospital**			
Yes	34(38.6)	37(21.0)	92(34.8)
No	54(61.4)	139(79.0)	172(65.2)
**Reason for delay in decision making to come to the hospital**			
Underestimating the severity of the condition	13(23.6)	14(37.8)	27(29.3)
Failure to understand the problem	35(63.6)	4(10.8)	39(42.4)
Bad experience with health facilities	6(10.9)	8(21.6)	14(15.2)
Lack of decision to go to the health facility	1(1.8)	9(24.3)	10(10.9)
Believed that God was in control	0(0)	2(5.4)	2(2.2)
**Means of transportation**			
Ambulance	54(61.4)	39(22.2)	93(35.2)
Public transport	23(26.1)	114(64.8)	137(51.9)
Private transport	11(12.5)	23(13.1)	34(12.9)
**Once you decide to go to the hospital, did you go striate away**			
Yes	62(70.5)	161(91.5)	223(84.5)
No	26(29.5)	15(8.5)	41(15.5)
**A reason not to go straight away to the hospital**			
Lack of transport	16(61.5)	12(80.0)	28(68.2)
Lack of money for transport	4(15.4)	0(0)	4(9.8)
Bad road condition	6(23.1)	3(20.0)	9(22.0)
**Time is taken to reach the health facility**			
≤ 10 KM	20(22.7)	137(77.8)	157(59.5)
>10KM	68(77.3)	39(22.2)	107(40.5)
**The time you wait to get the health provider at the health facility**			
≤ 30 min	80(90.9)	174(98.9)	254(69.2)
>30 min	8(9.1)	2(1.1)	10(3.8)
**Type of problem you faced in the hospital**			
Delay in making the diagnosis	3(3.4)	6(3.4)	9(3.4)
No assessment by the senior health care provider	2(2.3)	3(1.7)	5(1.9)
Lack of supply and equipment	2(2.3)	17(9.7)	19(7.2)
Poor monitoring of patients	10(11.4)	19(10.8)	29(11)
No problem faced	71(80.7)	131(74.4)	202(76.5)

### Determinants of maternal near-miss

In the bivariable logistic regression analysis model, residence, age, mode of admission, educational status, occupation, antenatal care, history of pregnancy-related complication, mode of delivery, medical disorder during pregnancy, and the first delay (delay to decide to come to the hospital) had a *p*-value of less than 0.25. In multivariable logistic regression analysis, age of the mother, educational status of women and husband, antenatal care, medical disorders during pregnancy, mode of admission, and mode of delivery were significant determinants of maternal near-miss.

Women with the age range of 25–34 years had 74% less risk of near-miss as compared to women with an age of greater than 35 years (AOR = 0.26, 95% CI: 0.09–0.75). Women who attended primary and secondary school (AOR = 4.80, 95% CI: 1.78–12.90), and women whose husbands had no formal education (AOR = 5.26, 95% CI: 1.46–18.90) were five times more likely to develop near-miss compared to those women who attended above secondary school. Referred cases had a five times higher risk of developing near-miss than self-admitted (AOR = 4.73, 95% CI: 1.78–12.55). Similarly, women who had no antenatal care follow-ups were three times more likely to develop near-miss compared to their counterparts (AOR = 2.75, 95% CI: 1.13–6.72). Besides, maternal near-miss was four times higher among women who delivered by cesarean section (AOR = 3.70, 95% CI: 1.42–9.60). Further, women who had medical disorders during pregnancy were 12.06 times more likely to develop maternal near-miss than women who had no medical conditions (AOR = 12.06, 95% CI: 2.82–51.55) (Table 5).

**Table 5:** Determinants of maternal near-miss in public hospitals, North Shewa Zone, Ethiopia, 2020 (N = 264).

**Variable**	**Cases**	**Control**	**COR (95% CI)**	**AOR (95% CI)**
	***n* (%)**	***n* (%)**		
**Age**				
15–24 years	13(14.8)	30(17.0)	0.49 (0.20–1.18)	0.62 (0.14–2.65)
25–34 years	54(61.3)	122(69.4)	0.50 (0.26–0.98)	0.26 (0.09–0.75)^*^
≥35 years	21(23.9)	24(13.6)	1	1
**Residence**				
Urban	26(29.5)	74(42.0)	1	1
Rural	62(70.5)	102(58.0)	1.73 (1.001–2.99)	0.71 (0.27–1.86)
**Education of women**				
No formal education	36(40.9)	29(16.5)	7.37 (3.62–14.99)	1.14 (0.31–4.14)
Primary & Secondary	35(39.8)	46(26.1)	4.52 (2.29–8.88)	4.80 (1.78–12.9)^*^
Above secondary	17(19.3)	101(57.4)	1	1
**Education of husband**				
No formal education	39(45.3)	23(13.1)	8.56 (4.20–17.5)	5.26 (1.46–18.90)^*^
Primary & secondary	28(32.6)	49(29.2)	2.88 (1.46–5.68)	3.90 (1.40–10.84)^*^
Above secondary	19(22.1)	96(57.1)	1	1
**Mother occupation**				
Housewife	42(47.7)	32(19.9)	7.96 (4.19–15.19)	1.92 (0.74–4.96)
Merchant	9(10.3)	19(10.8)	2.29 (0.87–6.02)	1.57 (0.38–6.02)
Employee	37(42.0)	122(69.3)	1	1
**Husband occupation**				
Farmer	39(45.3)	20(11.9)	3.95 (2.21– 7.06)	1.36 (0.35–5.18)
Merchant	17(19.8)	26(15.5)	1.56 (0.65–3.74)	0.58 (0.18–1.83)
Employee	30(34.9)	122(72.6)	1	1
**Mode of admission**				
Self	45(51.1)	144(81.8)	1	1
Referral	43(48.9)	32(18.2)	4.30 (2.43–7.58)	4.73 (1.78–12.55)^*^
**Antenatal care**				
Yes	55(62.5)	148(84.1)	1	1
No	33(37.5)	28(15.9)	3.17 (1.75– 5.72)	2.75 (1.13–6.2)^*^
**History of pregnancy–related complication**				
Yes	19(23.8)	18(11.8)	2.31 (1.13–4.72)	2.38 (0.83–6.80)
No	61(76.2)	134(88.2)	1	1
**Mode of delivery**				
SVD	62(77.5)	138(90.8)	1	1
C/S	18(22.5)	14(9.2)	2.86 (1.33–6.11)	3.7 (1.42–9.60)^*^
**Medical disorders during pregnancy**				
Yes	14(15.9)	8(4.5)	3.97 (1.59–9.87)	12.1(2.82–51.55)^*^
No	74(84.1)	168(95.5)	1	1
**Delay to decide to come to the hospital**				
Yes	34(38.6)	37(21.0)	2.36 (1.34–4.14)	0.46 (0.17–1.22)
No	54(61.4)	139(79.0)	1	1

* Statistically significant at p–value ≤ 0.05 SVD, Spontaneous vaginal delivery; C/S, Cesarean section.

## Discussion

Maternal near-miss remains a significant public health problem in developing countries. The study was conducted to identify determinants of maternal near-miss in North Shewa Zone public hospitals. Accordingly, age, educational status, antenatal care visit, medical disorder during pregnancy, mode of delivery, and mode of admission were determinants of maternal near-miss.

Women aged 25–34 years were less likely to develop maternal near-miss (MNM) (AOR = 0.26, 95% CI: 0.09–0.75). This finding was consistent with the previous studies conducted in Brazil (18) and Nigeria (19). The study found a proportional relationship between higher maternal near-miss ratio, maternal mortality ratio, and advancing age (particularly over 35 years). This might be because several medical disorders that complicate pregnancy, i.e., hypertension, diabetes, heart, and thyroid disease, are typical in older women.

Low maternal education also significantly increases the odds of maternal near-miss (AOR = 4.80, 95% CI: 1.78–12.90). Similarly, women whose husbands had lower educational status had a high risk of developing maternal near-miss (AOR = 5.26, 95% CI: 1.46–18.90). This finding is in line with the studies conducted in Southwestern Nigeria (19), West Arsi (20), and the Tigray region (16), where low educational status increases the odds of maternal near-miss. This could be due to education increasing access to information, which is associated with better knowledge of their health, obstetric complications, and improved decision-making capabilities. These positively influence the health-seeking behavior of the mother. In contrast, a prospective study in Iran indicated that maternal near-miss was significantly associated with higher educational status (21). This could be due to women with higher academic levels might prefer to undergo a cesarean section (CS), which may ultimately increase the probability of near-miss events. In our setting, this is very unlikely where most women, irrespective of their educational level, have a solid opposition to CS.

Mothers who did not have antenatal follow-ups were more likely to experience near-misses (AOR = 2.75, 95% CI: 1.13–6.72). This finding was supported by prior studies done in Morocco (22), Western Ethiopia (23), the Tigray region (16), and West Arsi (20). Besides, in Nigeria, attending antenatal care was a protective factor for maternal near-miss (24). ANC visits given to pregnant mothers are very important for monitoring pregnancy and early identification and management of maternal complications. Besides, antenatal care visits increase mothers' awareness about anticipating labor and its signs, better sites for delivery, and the importance of timely seeking care rather than managing at home. However, according to the Ethiopian demographic and health survey 2019, only 43% of women received four or more ANC visits from a skilled provider (9).

In line with previous studies conducted in Southern Ethiopia (25), the Tigray region (16), and West Arsi (20), this study also found that women with a prior history of cesarean section (CS) were more prone to maternal near-miss events (AOR = 3.70, 95% CI: 1.42–9.60). A prospective cohort study in Northeastern Brazil also indicated that cesarean section was significantly associated with the occurrence of maternal near-misses (26). Despite saving the life of a woman and newborn, prior history of cesarean section raises the risk of hemorrhage, recurrence, placenta accrete in scar tissue, thromboembolism, a uterine rupture in attempted vaginal birth after CS, and other complications, which could increase the probability of MNM. To minimize unnecessary risks for the women, cesarean section, particularly elective non-medical CS should be reduced to the acceptable level recommended by WHO (10-15%) (27), and the misconception “once a cesarean section, always a cesarean section” should be avoided.

The odds of maternal near-miss were significantly higher among women with medical disorders during pregnancy (AOR = 12.06, 95% CI: 2.82–51.55). Several studies have documented that chronic medical diseases happening during pregnancy increase the risks of MNM (16, 20, 25). Similar findings were also reported from Accra, Ghana, in which women with chronic medical disorders were more likely to experience maternal near-miss (28). Chronic medical condition, i.e., anemia, diabetes, cardiovascular disease, and chronic hypertension substantially raises the risk of preterm delivery, pre-eclampsia, intrauterine growth retardations, placental abruption, and other complications. Encouraging pregnant mothers to screen for non-communicable diseases during antenatal care visits would be an excellent step toward reducing the incidence of maternal near-misses.

This study found that the admission status of women was a significant determinant of MNM (AOR = 4.73, 95% CI: 1.78–12.55). Women who were referred from other facilities had a five-fold risk of developing maternal near-miss. A similar result was also represented in Nigeria (19) and South Ethiopia (17). The possible reason might be that complicated cases are more likely to be referred, and lack of transport or too long distance to reach referral facilities (second delay), delayed referrals, and lack of early detection of potentially life-threatening complications would contribute to maternal near-miss events. Improving the accessibility of maternal health care to the most nearby health facility, improving the referral system, and access to road and other transport facilities would reduce maternal near-miss.

## Limitation

Recall bias is possibly introduced during data collection. Respondents would have a problem with memorizing some of the variables happening before, introducing a recall bias.

## Conclusion and recommendation

Age, educational level, antenatal care follow-ups, medical disorder during pregnancy, mode of admission, and mode of delivery were significant predictors of maternal near-miss. Socio-demographic development, use of ANC services, early detection and management of medical diseases, reducing cesarean section, and improving the referral systems are crucial to minimizing the maternal near-miss. Besides, the zonal health departments would improve access to maternal health care services for all mothers at the lowest health facilities.

## Data availability statement

The raw data supporting the conclusions of this article will be made available by the authors, without undue reservation.

## Ethics statement

The studies involving human participants were reviewed and approved by Debre Berhan University, College of Medicine and Health Science. The patients/participants provided their written informed consent to participate in this study.

## Author contributions

HNT, ETA, SH, OY, and GBM conceived the study, performed the analysis, and interpreted and drafted the manuscript. HNT, ETA, SH, OY, MT, and GBM contributed to the interpretation of data, drafting the manuscript, and critically reviewing the manuscript. All authors read and approved the final manuscript.

## Conflict of interest

The authors declare that the research was conducted in the absence of any commercial or financial relationships that could be construed as a potential conflict of interest.

## Publisher's note

All claims expressed in this article are solely those of the authors and do not necessarily represent those of their affiliated organizations, or those of the publisher, the editors and the reviewers. Any product that may be evaluated in this article, or claim that may be made by its manufacturer, is not guaranteed or endorsed by the publisher.

## Abbreviations

ANC: Antenatal Care
AOR: Adjusted Odds Ratio
CI: Confidence Interval
COR: Crude Odds Ratio
CS: Cesarean Section
EC: Ethiopian Calendar
EDHS: Ethiopia Demographic Health Survey
MNM: Maternal Near Miss
NASG: Non-pneumatic Anti Shock Garment
SDG: Sustainable Development Goal
WHO: World Health Organization.
